# Lachesis and Lycopodium in Diphtheria

**Published:** 1882-11

**Authors:** E. J. Lee


### LACHESIS AND LYCOPODIUM IN DIPHTHERIA.

In his excellent little work on Diphtheria, Dr. Gregg lays the greatest stress, as an indication for the proper remedy, upon the side of the throat upon which the disease first appears. If on the right side, give Lycopodium; if on the left side, Lachesis. Lachesis, he says, may be indicated, especially in very malignant cases, even if the disease does begin on the right side; especially if Lyc. appears to have been indicated and fails. On the other hand, if Lachesis appears to be indicated, yet fails, he advises that Apis be studied. It is well for us not to rely upon *one* symptom in prescribing for any disease; the more handles we have the surer is our hold.

As an aid to a diagnosis between Lachesis and Lycopodium, we give a few differential points noted by by Dr. Hering: "In diphtheria, when both remedies are of the greatest importance, the choice often decides for Lachesis by aggravation from swallowing saliva, not food; for Lycopodium, if worse when swallowing warm drinks (Raue); Lachesis has more exudative patches on the tonsils, particularly on the *left* side; Lycopodium, a darkish hue, on the fauces, particularly on the *right* side; with both remedies the patients are worse after sleep, Lachesis particularly in the morning (siesta), with Lycopodium when awaking after every nap (children are cross and naughty, kick about). Other characteristics of Lachesis are oversensibility of the throat to touch [Apis also], or croup-like symptoms; of Lycopodium the breathing through the mouth, or the dilatation of the nostrils with every inspiration, or parotid swellings."

